# The Investigation of Lipoxygenases as Therapeutic Targets in Malignant Pleural Mesothelioma

**DOI:** 10.1007/s12253-019-00652-x

**Published:** 2019-04-02

**Authors:** Lily Oguh-Olayinka, Vijay Agarwal, Dulani Ranatunge, Anne Campbell, Stefan Laufer, Lynn Cawkwell, Michael J. Lind

**Affiliations:** 1 Research Laboratories, Hull York Medical School, Daisy Building, Castle Hill Hospital, Hull, HU16 5JQ UK; 2 Queens Centre for Oncology and Haematology, Hull and East Yorkshire NHS Trust, Hull, UK; 3 Histopathology Department, Hull and East Yorkshire NHS Trust, Hull, UK; 4 Department of Pharmaceutical Chemistry, Eberhard Karls University, Tübingen, Germany; 5 Department of Biomedical Science, University of Hull, Hull, UK

**Keywords:** Arachidonic acid, Cyclooxygenase, Immunohistochemistry, Lipoxygenase, Mesothelioma

## Abstract

Advanced malignant pleural mesothelioma (MPM) has an extremely poor prognosis with limited chemotherapy options, therefore the identification of new therapeutic targets would aid in disease management. Arachidonic acid is metabolised by cyclooxygenase and lipoxygenase enzymes. The lipoxygenase isoenzymes 5-LOX and 12-LOX have been implicated in carcinogenesis. We aimed to examine 5-LOX and 12-LOX protein expression in a large retrospective series of mesothelioma samples. Further to this, the in vitro cytotoxic effects of lipoxygenase pathway inhibitors were investigated in mesothelioma cells. Archival samples from 83 patients with MPM were examined by immunohistochemistry for expression of the 5-LOX and 12-LOX proteins. The MTS assay was used to assess cell viability following 72 h treatment with the lipoxygenase pathway inhibitors baicalein, licofelone, MK-886 and zileuton in the MPM cell lines NCI-H2052, NCI-H2452 and MSTO-211H. Positive 12-LOX protein expression was recorded in 69/83 (83%) and positive 5-LOX expression was observed in 56/77 (73%) of MPM tissue samples. Co-expression of 5-LOX with 12-LOX was seen in 46/78 (58%) of MPM samples. Positive expression of 5-LOX, 12-LOX and COX-2 proteins was identified in the NCI-H2052, NCI-H2452 and MSTO-211H MPM cell lines. Baicalein (12-LOX and 15-LOX inhibitor) was effective in 3/3 MPM cell lines at low concentrations with an IC50 range of 9.6 μM to 20.7 μM. We have demonstrated that the 5-LOX and 12-LOX proteins are expressed in a significant proportion of MPM samples (73% and 83% respectively) and may represent novel therapeutic targets in this disease. We have demonstrated that the inhibition of the LOX pathway using baicalein may be effective as a novel treatment for MPM, however further human pharmacokinetic studies are required in order to establish whether the concentration used in vitro is clinically achievable.

**Electronic supplementary material:**

The online version of this article (10.1007/s12253-019-00652-x) contains supplementary material, which is available to authorized users.

## Introduction

Malignant pleural mesothelioma (MPM) is a rare, aggressive tumour. Despite recent advances in chemotherapy, it is associated with a universally poor prognosis. In 80% of cases, MPM can be attributed to asbestos fibre exposure with a median latency of at least 32 years [1, 2]. Histologically MPM can be classified into epithelial, biphasic and sarcomatoid subtypes. The epithelial subtype is associated with longer survival when compared with sarcomatoid [3, 4]. With the administration of cisplatin/pemetrexed the median survival of MPM is still only 12 months and currently there is no widely approved second line regimen after failure of first line treatment [5–8].

Arachidonic acid is metabolised by lipoxygenase (LOX) enzymes to form leukotrienes (LTs) and by cyclooxygenase (COX) enzymes to form prostanoids, including prostaglandin E_2_ (PGE_2_) which has been implicated in inflammation and carcinogenesis [9–13]. COX exists in two forms, COX-1 and COX-2. COX-2 is overexpressed in a wide variety of tumours and this feature has been correlated with the malignant properties of cancers. Inhibition of COX-2 has been reported to reverse malignant behaviour such as antiapoptosis, angiogenesis and invasion [14, 15] and epidemiological evidence suggests that regular use of COX-2 inhibitors may reduce the risk of several cancers [16]. We have previously shown using immunohistochemistry (IHC) that COX-2 is overexpressed in MPM and that the specific COX-2 inhibitor DuP-697 can potentiate the in vitro cytotoxic effects of pemetrexed in MPM cell lines [4, 17]. Functional interactions between COX-2 and LOX enzymes have been identified [18] and, of the three known LOX isoenzymes (5-LOX, 12-LOX and 15-LOX), 5-LOX and 12-LOX have also been implicated in carcinogenesis [13, 19]. The 5-LOX enzyme interacts with 5-LOX activating protein (FLAP) and converts arachidonic acid into LTA4. LTA4 can be converted into 5-hydroxyeicosatetraenoic acid (HETE) or can be hydrolysed into LTB4 or LTC4 [12, 20]. LTB4 has been shown to cause cell proliferation and survival via its action on the ERK pathway in colon cancer cell lines and via the PI3K-AKT and MAPK pathways in pancreatic cancer cell lines [21, 22]. Arachidonic acid is converted into 12-HETE by its interaction with 12-LOX [20]. The 12-HETE enzyme interacts with the NFkB pathway, resulting in an antiapoptotic effect, in prostate cancer cells [23] and may interact with various growth factors resulting in angiogenesis, invasion and metastasis [24]. The expression of 5-LOX and 12-LOX has been associated with carcinogenesis in various solid tumours. Hong and colleagues reported the first evidence of the potential role for 5-LOX in cancer through the demonstration of expression of 5-LOX and FLAP transcripts in different epithelial cancer cell lines [25]. Increased expression of 5-LOX and LTB4 transcripts was later demonstrated in human pancreatic cancer cells, when compared with cultivated normal pancreatic ductal cells [26] and a 6-fold upregulation of 5-LOX transcripts was demonstrated in prostate cancer tissue, when compared with a matched benign tissue sample [27] The overexpression of 5-LOX protein was demonstrated in 22 matched samples of benign and malignant tissue obtained from patients with prostate cancer, when assessed by immunoblotting and IHC [27]. This immunoblotting analysis demonstrated overexpression of 5-LOX protein in 73% of samples with a 2.6 fold greater expression in the malignant tissue and the immunohistochemical analysis confirmed the up-regulation of 5-LOX protein in malignant tissue [27]. Further studies on the overexpression of LOX enzymes have since been documented in other epithelial cancers, including bladder [28], breast [29], colon [30], melanoma [31], oesophagus [32, 33], and oral [34]. The role of LOX enzymes in MPM has not been widely studied. 5-LOX transcripts were demonstrated to be upregulated in MPM cell lines, when compared with normal mesothelial cells, whilst the expression of 12-LOX transcripts was detected in both normal and malignant cells [35]. We therefore aimed to investigate the importance of 5-LOX and 12-LOX protein expression in MPM and benign pleura tissue samples and to study the effects of in vitro inhibition of the arachidonic acid pathway in MPM cell lines.

## Materials and Methods

### Archival Tissue Samples

Research Ethics Committee approval was granted for the study (ref 11/00/212). Archival MPM tissue samples were obtained from 83 patients diagnosed between 1992 and 2000 at Hull Royal Infirmary, UK. The majority of patients were male (76/83; 92%). The original diagnostic histology slides were reviewed by a histopathologist specialising in MPM (AC) and clinicopathological details for all samples were available. There were 44/83 (53%) epithelial, 25/83 (30%) biphasic and 14/83 (17%) sarcomatoid histological subtypes. In order to investigate benign pleural tissue, 8 archival samples were obtained which were derived from male patients who had undergone thoracoscopic intervention to prevent recurrence of spontaneous pneumothorax. These 8 samples were classified histologically by a histopathologist (AC) into non-reactive (*n* = 2), mildly reactive (n = 2) and very reactive (*n* = 4) based on the reactivity of the mesothelial cells.

### Immunohistochemistry

The 83 archival MPM tissue samples and 8 benign pleural tissue samples were analysed by IHC as previously described [36] with minor modifications. In brief, endogenous peroxidase enzymes were blocked and antigens were heat-retrieved by boiling under pressure for 3 min at 15 psi in 1:100 Antigen Unmasking Solution (H-3300, Vector Laboratories Inc.) within a stainless steel pressure cooker. Non-specific staining was blocked using normal horse serum (#PK-7800, Vector Laboratories Inc.) and endogenous biotin and avidin binding sites were also blocked (#SP-2001, Vector Laboratories Inc.). Incubations with anti 5-LOX (#ab169755, Abcam) or anti 12-LOX (#ab23678, Abcam) antibodies were performed using a dilution of 1:100 for 2 h. Negative (antibody-omitted) control and positive control slides, which consisted of archival colorectal cancer tissue, were included in each batch. Antibody localisation was detected and visualised using a streptavidin/peroxidase method (#PK-7800, Vector Laboratories Inc.) with DAB as chromogen. Slides were counterstained with haematoxylin and independently reviewed by 3 of the co-authors, including a histopathologist specialising in MPM (AC). Discordant scores were reviewed in open discussion. For MPM samples, “positive” protein expression was recorded if there was moderately strong staining in more than 25% of the malignant cells and “negative” protein expression was recorded if no, or only weak, staining was seen or if staining was seen in less than 25% of the malignant cells.

### Statistical Analysis

Statistical analysis was performed using SPSS software version 19.0 (SPSS, Chicago, USA). Univariate analysis was carried out for 5-LOX and 12-LOX expression using Kaplan Meier survival curves with log rank analysis. Multivariate analysis was calculated using Cox regression analysis to take into account the histological subtypes which are known to be an independent prognostic variable in MPM [4].

### Cell Lines

The MPM cell lines NCI-H2452, NCI-H2052 and MSTO-211H were obtained from the American Type Culture Collection (ATCC). In addition, the non-small cell lung cancer (NSCLC) cell line A549 was obtained from the European Collection of Cell Cultures (ECACC). Cells were grown in RPMI 1640 medium supplemented with 2 mM L-glutamine, 10% (*v*/v) foetal bovine serum, 100 U/ml penicillin and 100 μg streptomycin in a humidified incubator with 5% CO_2_ at 37 °C. Cell lines were passaged at 70–80% confluence and regularly checked for mycoplasma contamination.

### Cell Lysis and Immunoblotting

Cells were grown to 70–80% confluence in then lysed in Laemmli buffer (65 mM Tris-HCl pH 6.8, 10% glycerol, 2% SDS, 0.001% bromophenol blue) with the addition of 5% β-mercaptoethanol and 1% protease inhibitor mix (#80–6501-23, Amersham Biosciences). Protein lysates were quantified using the RCDC protein assay (# 500–0122, Biorad) and 50 μg of protein was analysed per lane on a 12% acrylamide gel (#25222, Pierce) under reducing conditions and transferred to a nitrocellulose membrane using a semi-dry iBlot system (Life Technologies). Membranes were blocked in 5% non-fat milk before samples were probed for 2 h with the anti 5-LOX antibody (#ab39347, Abcam) at a final concentration of 1:250 and the anti 12-LOX antibody (#ab23678, Abcam) at a final concentration of 1:500. To serve as a loading control, the anti α-tubulin antibody (#ab7291, Abcam) was applied at 1:3000 for 2 h. Visualisation of protein bands was achieved using the SuperSignal West Pico Chemiluminescent Substrate kit (#34078, Pierce).

### Cell Cytotoxicity Assay

Commercially available inhibitors were purchased as follows: zileuton (#3308, Tocris Bioscience), which is a 5-LOX inhibitor [37]; MK-886 (#1311, Tocris Bioscience), which is a FLAP inhibitor [38]; baicalein (#1761, Tocris Bioscience), which is a 12-LOX and 15-LOX inhibitor [39]; celecoxib (#3786, Tocris Bioscience), which is a selective COX-2 inhibitor [40]. In addition, licofelone (ML3000) was provided as a gift by Professor Stefan Laufer (Department of Pharmaceutical Chemistry, Eberhard Karls University, Tübingen, Germany). Licofelone is a dual COX/5-LOX inhibitor [41]. Stock drug solutions were prepared in DMSO and stored at -20 °C for further use. Drugs were diluted in fresh media prior to each experiment. Cells were plated in 96-well plates at 1 × 10^3^ cells/well and grown overnight in supplemented media as above. After 24 h, cells were treated in replicates of 6 and cell viability was measured after 72 h using the CellTiter 96 Aqueous One Solution Cell Proliferation Assay (#G3581, Promega). Following the 3 h labeling of metabolically active cells with MTS, results were measured at 492 nm using an absorbance plate reader (Multiskan FC Microplate photometer, Thermo Scientific). Values were normalised to untreated control cells in order to generate dose response curves. At least 3 independent experiments were carried out for each drug analysis before IC50 values were calculated using GraphPad Prism 5.0 software. Student’s paired t test was used to assess the differences observed between single agent treatment and combinations (*p* < 0.05). To assess drug synergy between celecoxib and baicalein on cell growth inhibition of the cell lines, a combination index (CI) was calculated using data obtained from the MTS assay. Concentration-effect curves were generated as a plot of the fraction of unaffected cells versus drug concentration in accordance with the Chou-Talalay method [42] using the following CI equation: CI = (D1)/(D1a) + (D2)/(D2a) + (D1 x D2)/(D1a x D2a), where D1 and D2 are the concentrations of baicalein and celecoxib respectively that exhibited a determined effect when applied simultaneously to the cells and D1a and D2a are the concentrations of these drugs that exhibited the same determined effect when used as single agents. The CI values indicate a synergistic effect when <1, an additive effect when equal to 1 and an antagonistic effect when >1 [42].

## Results

### Expression of 12-LOX Protein

**Fig. 1:**
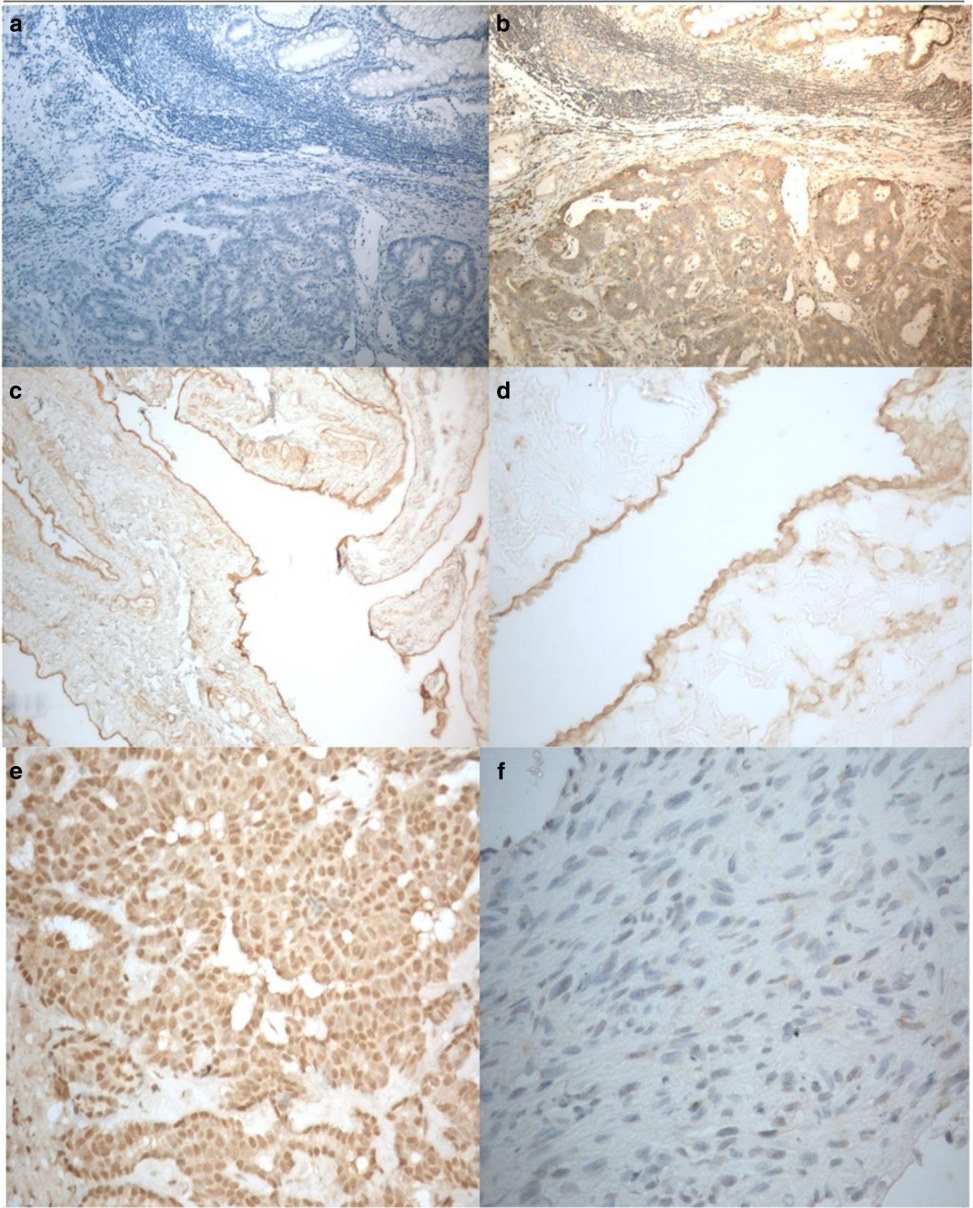
**Expression of 12-LOX protein demonstrated by IHC using primary antibody ab23678 (Abcam)**. **a** Negative control (antibody omitted) (× 100). **b** Positive control (colorectal cancer with adjacent normal mucosa, × 100). **c**, **d** 12-LOX expression in non-reactive benign pleural tissue (× 100 and × 400 respectively). **e** Epithelial subtype of MPM demonstrating strong positive expression (× 400). **f** Biphasic subtype of MPM demonstrating negative expression (× 400)

All 8/8 benign pleural samples exhibited similar nuclear and cytoplasmic staining patterns, irrespective of their reactive status (Fig. 1). In the MPM samples the expression of the 12-LOX protein was again predominantly found in the nucleus and cytoplasm of the malignant cells, with varying intensity (Fig. 1). Positive 12-LOX protein expression was recorded in 69/83 (83%) of MPM tissue samples (Table 1). The expression of 12-LOX was not associated with survival (*p* = 0.455).

**Table 1:** Immunohistochemical analysis of 5-LOX, 12-LOX and COX-2 protein expression categorised by histological subtype

	Total	Epithelial	Biphasic	Sarcomatoid
12-LOX positive	69/83 (83%)	38/44 (86%)	21/25 (84%)	10/14 (71%)
5-LOX positive	56/77 (73%)	30/42 (71%)	18/22 (81%)	8/13 (62%)
COX-2 positive^a^	58/93 (62%)	37/48 (77%)	15/27 (56%)	6/18 (33%)

^a^Data for this cohort as previously reported ^4^

### Expression of 5-LOX Protein

**Fig. 2:**
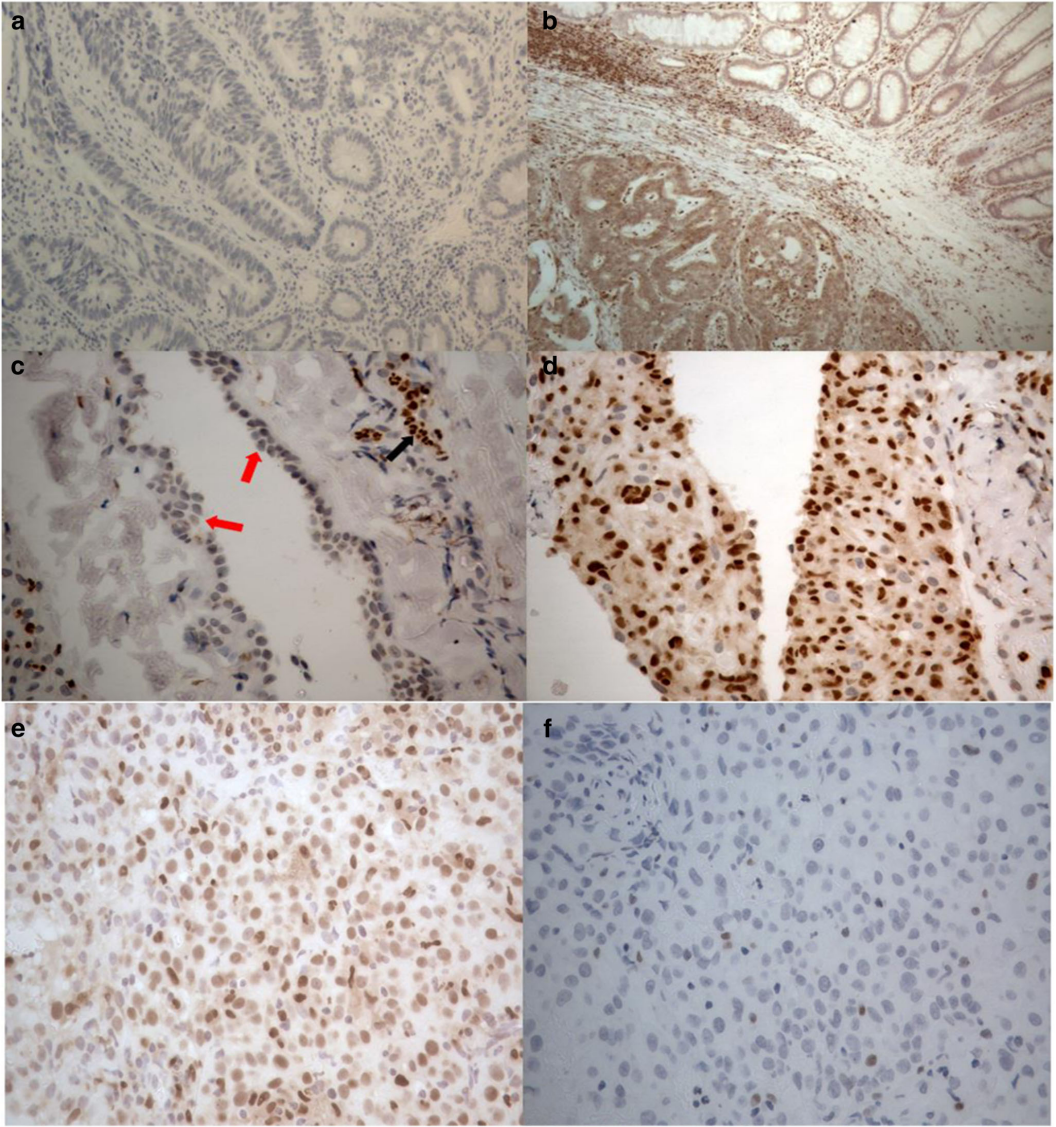
**Expression of 5-LOX protein demonstrated by IHC using primary antibody ab169755 (Abcam)**. **a** Negative control (antibody omitted) (× 100). **b** Positive control (colorectal cancer with adjacent normal mucosa, × 100). **c** Benign pleural tissue with non-reactive mesothelial cells, which can be seen as an organised strip of mesothelial cells on the surface (red arrows), exhibiting no expression of 5-LOX protein. Inflammatory cells (black arrow) can be seen in the connective tissue (× 400). **d** Benign pleural tissue with reactive mesothelial cells and inflammatory cells demonstrating positive expression for 5-LOX (× 400). **e** Epithelial subtype of MPM demonstrating positive expression (× 400). **f** Epithelial subtype of MPM demonstrating negative expression (× 400)

The 8 benign pleural samples exhibited differential expression of the 5-LOX protein based on their reactive status (Fig. 2). Where staining was observed, 5-LOX protein was localised to the cytoplasm and nucleus. Few inflammatory cells were seen in the 2 non-reactive pleural samples and the mesothelial cells exhibited no expression of 5-LOX protein. Mildly reactive pleural samples (*n* = 2) exhibited weak staining for 5-LOX in the mesothelial cells and very reactive pleural samples (*n* = 4) exhibited strong positive staining for 5-LOX in the mesothelial cells (Fig. 2).

Of the 83 MPM tissue samples, 77 samples were successfully scored. The immunohistochemical staining revealed nuclear and cytoplasmic expression of the 5-LOX protein in malignant cells with varying intensity (Fig. 2). Positive staining of lymphocytes and other inflammatory cells served as an internal positive control. Positive 5-LOX expression was observed in 56/77 (73%) of MPM tissue samples (Table 1). Overall, the expression of 5-LOX was not associated with survival (*p* = 0.640), however when considering only the sarcomatoid subtype the positive expression of 5-LOX was significantly associated with improved survival (median survival 4.2 months versus 1 month in 5-LOX negative cases; *p* = 0.028) (online resource 1).

### Correlations Between 5-LOX, 12-LOX and COX-2 Expression

Co-expression of 5-LOX with 12-LOX was seen in 46/78 (58%) of the MPM samples but this was neither statistically significant nor associated with survival. We have previously published the COX-2 protein expression data for this cohort [4] (Table 1). A total of 41/77 (53%) of samples demonstrated co-expression of COX-2 with 5-LOX proteins and this status was significantly associated with improved survival when compared with cases which were negative for both proteins (median survival 8.7 months versus 2.2 months; *p* = 0.011) (online resource 1). Co-expression of COX-2 with 12-LOX was seen in 47/83 (56%) of the samples. In univariate analysis cases demonstrating a COX-2 positive /12-LOX negative status or co-expression of COX-2 with 12-LOX were associated with longer survival (online resource 1).

### Expression 5-LOX and 12-LOX in MPM Cell Lines

**Fig. 3:**
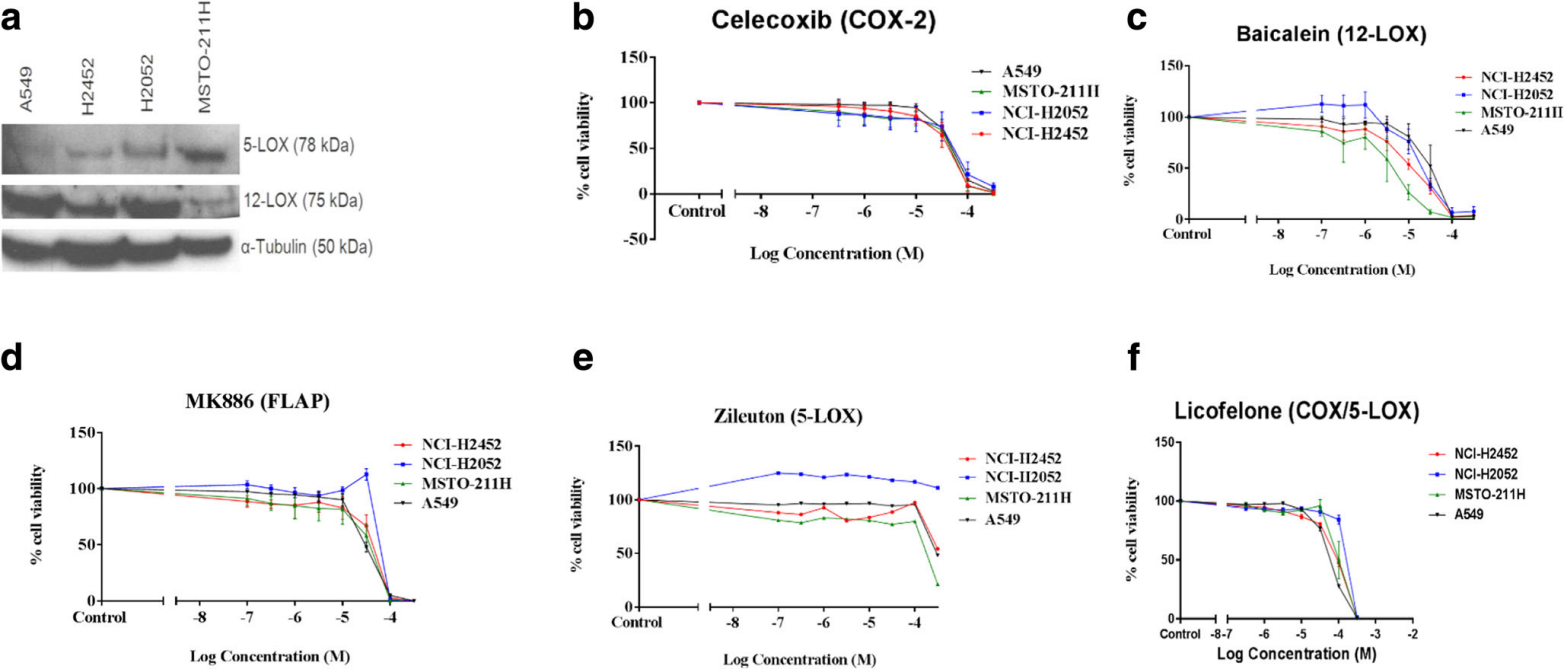
**Effect of COX-2, LOX and FLAP inhibitors on the viability of MPM cells**. **a** Immunoblotting analysis of the NSCLC cell line A549 and the MPM cell lines NCI-H2452, NCI-H2052 and MSTO-211H. **b**-**f** Cell proliferation (MTS) assays to investigate the single-agent effect in MPM and A549 cells of a COX-2 inhibitor (celecoxib), a 12-LOX/15-LOX inhibitor (baicalein), a FLAP inhibitor (MK-886), a 5-LOX inhibitor (zileuton) and a dual COX/LOX-5 inhibitor (licofelone) respectively. Following treatment for 72 h, cell viability was determined using the MTS reagent and expressed as a ratio of cell viability in comparison to the relevant control (cells treated with <0.1% dimethyl sulfoxide). Each data point is the mean of 18 replicates and error bars represent the standard error of the mean. The IC50 values generated are shown in Table 2

The protein expression of 5-LOX and 12-LOX in the MPM cell lines is shown in Fig. 3 and the relative expression levels following normalisation, are summarised in Table 2. We have previously reported the expression of COX-2 in these cell lines [4]. Positive expression of 5-LOX, 12-LOX and COX-2 proteins was identified in all of the MPM cell lines and the NSCLC cell line A549.

**Table 2:** Immunoblotting analysis of the COX-2, 5-LOX and 12-LOX proteins. The relative expression, following normalisation of the bands shown in Fig. 3(a), is summarised for 3 MPM cell lines (NCI-H2452, NCI-H2052, MSTO-211H) and the NSCLC cell line A549

Cell line	COX-2^a^	5-LOX	12-LOX
A549	++	++	++
NCI-H2452	+++	++	++
NCI-H2052	++	+++	++
MSTO-211H	++	++	+

^a^Data previously reported for this cell line panel ^4^

### Effect of LOX Pathway Inhibitors on Cell Viability

Cell viability was determined in all cell lines following single-agent treatment for 72 h with increasing concentrations of the LOX pathway inhibitors baicalein, MK-886, zileuton and licofelone, in addition to the COX-2 inhibitor celecoxib (Fig. 3, Table 3). Celecoxib (COX-2 inhibitor) demonstrated similar anti-proliferative effects in all MPM cell lines with an IC50 range of 39.2 μM to 48.1 μM. At low concentrations, zileuton (5-LOX inhibitor) and licofelone (dual COX/5-LOX) inhibitor did not demonstrate an effect in any of the MPM cell lines. MK-886 (FLAP inhibitor) exerted an effect at low concentrations in 2/3 of the MPM cell lines, however baicalein (12-LOX and 15-LOX inhibitor) was effective in 3/3 MPM cell lines at low concentrations with an IC50 range of 9.6 μM to 20.7 μM.

**Table 3:** The effect of arachidonic acid pathway inhibitors on MPM cells. As shown in Table 2, all cell lines demonstrated expression of 5-LOX, 12-LOX and COX-2 proteins. The IC50 values, calculated from the data shown in Fig. 3, are shown for the MPM cell lines (NCI-H2452, NCI-H2052, MSTO-211H) and the NSCLC cell line A549. For reference, the published human Cmax range for each inhibitor was converted into the equivalent molar concentrations. Baicalein, MK-886 and licofelone do not currently have a published recommended daily dose and therefore the clinically achievable Cmax may be higher than shown

	Celecoxib	Baicalein	MK-886	Zileuton	Licofelone
Inhibitor of: Published Cmax range:	COX-2 3.0–6.2 μM at the recommended daily dose of 400 mg per day ^56^	12-LOX & 15-LOX 0.1–0.7 μM ^55^	FLAP 4.0–8.7 μM ^57^	5-LOX 14.1–22.8 μM at the recommended daily dose of 2400 mg per day ^58^	Dual COX/5-LOX 2.9–6.2 μM ^59^
A549	47.9 μM	28.8 μM	29.7 μM	307.5 μM	58.6 μM
NCI-H2452	39.2 μM	10.7 μM	39.0 μΜ	342.7 μΜ	80.7 μΜ
ΝCΙ-Η2052	48.1 μΜ	20.7 μΜ	84.0 μΜ	579.2 μΜ	140.0 μΜ
ΜSΤΟ-211Η	42.2 μΜ	9.6 μΜ	30.5 μΜ	137.3 μΜ	99.7 μΜ

### Effect of Combined Use of COX-2 and LOX Pathway Inhibitors on Cell Viability

**Fig. 4:**
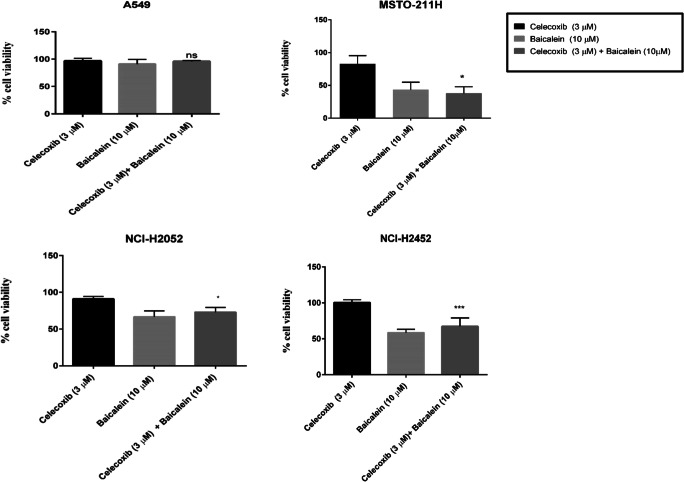
**Effect of combining celecoxib with baicalein on the viability of MPM cells**. Cell proliferation (MTS) assays were performed to investigate the combined effects of 3 μM celecoxib (COX-2 inhibitor) with 10 μM baicalein (12-LOX and 15-LOX inhibitor) in the NSCLC cell line A549 and the MPM cell lines NCI-H2452, NCI-H2052 and MSTO-211H. Following treatment for 72 h, cell viability was determined using the MTS reagent. The data represents the mean and standard deviation of six replicates from at least three independent experiments and the statistical significance of the combination versus celecoxib-alone is shown (ns – not significant; * *p* = 0.01 to 0.05; *** *p* = 0.0001 to 0.001). The Chou-Talalay combination index (3.38 in A549; 1.72 in NCI-H2452; 1.36 in NCI-H2052; 0.73 in MSTO-211H) indicates a synergistic effect of celecoxib and baicalein in the MSTO-211H cells at the selected doses

As a proof of principle, we investigated the effect of combining celecoxib and baicalein on cell viability using a clinically achievable concentration of celecoxib (3 μM; Table 3). A concentration of 10 μM was selected for baicalein based on the single-agent IC50 data for the MPM cells (Table 3). Cell viability was determined following combined treatment for 72 h (Fig. 4).

## Discussion

MPM is highly resistant to tyrosine kinase inhibitors and the preferred treatment remains the combination of a platinum-based compound and a folate antimetabolite [5, 7, 8]. There are currently no approved second-line treatments for patients who do not respond to the chemotherapy regimen indicating an urgent need for the development of more effective therapies and a better understanding of this malignancy. Previous studies have demonstrated the potential role of COX-2 as a therapeutic target in MPM [4, 43–45] and the selective COX-2 inhibitor celecoxib has been included, in combination with other drugs, in clinical trials for patients with MPM [46]. In the current study we have demonstrated that the 5-LOX and 12-LOX proteins are expressed in a significant proportion of MPM samples (73% and 83% respectively) and may represent novel therapeutic targets in this disease. The co-expression of COX-2 with 5-LOX was associated with improved survival in this cohort and, in the sarcomatoid subtype only, the expression of 5-LOX was associated with improved prognosis however these preliminary findings require further confirmation using a larger number of cases. The benign samples used in this study were from patients with an underlying condition which may lead to the presence of reactive mesothelial cells due to inflammation. 12-LOX expression was seen in non-reactive and reactive benign pleural tissue samples whilst 5-LOX expression was evident only in cases with reactive mesothelial cells. To our knowledge, this is the first study to investigate 5-LOX and 12-LOX protein expression in MPM tissue samples.

A panel of MPM cell lines, which exhibited expression of the COX-2, 5-LOX and 12-LOX targets, demonstrated no response to treatment for 72 h with the 5-LOX inhibitor zileuton at the clinically relevant concentration range. In each cell line, the IC50 for celecoxib was reached at a concentration which was outside the clinically relevant range. The maximum tolerated dose for baicalein, MK-886 and licofelone in humans is not available and therefore to further examine the in vitro effects of simultaneous inhibition of the COX-2 and LOX pathways we examined baicalein in combination with celecoxib, which was used at the clinically relevant concentration. Baicalein, which is known to inhibit 12-LOX as well as 15-LOX [39], had demonstrated an effect in all 3 MPM cell lines at relatively low concentrations as single agent when compared to the other LOX pathway inhibitors. Baicalein has been reported to reduce cell proliferation in vitro in other cancer cell lines at concentrations of 5–80 μM [47–50] and may induce cancer cell death via inhibition of CDC2 kinase and survivin [48]. There has only been one previous report of the baicalein treatment of MPM cells and no effect was seen when using a single fixed concentration of 2 μM [35]. The in vitro combination results indicated that celecoxib/baicalein treatment may be more effective than celecoxib alone in these cell lines, however 10 μM baicalein alone appears to be mainly responsible for this effect with drug synergy demonstrated in only 1 cell line.

In summary, this study has shown that 5-LOX and 12-LOX proteins are expressed at high frequency in mesothelioma samples and may represent therapeutic targets. The role of 15-LOX in MPM requires further study. We acknowledge that inhibitors used within this study have been previously reported to inhibit tumour cells independent of lipoxygenase inhibition, hence further studies on the effect of the inhibitors on PGE2 and LTB4 levels in conjunction with cell death in mesothelioma cells requires further evaluation [51–53]. 12-LOX has also been reported to be predominantly expressed in the cytoplasm however nuclear localization of 5-LOX and 12-LOX have been reported previously by [54, 55].

As a proof of principle, we have demonstrated that the inhibition of mesothelioma cells using baicalein may be effective as a novel treatment for MPM, however further human pharmacokinetic studies are required in order to establish whether the concentration used in vitro is clinically achievable since the single pharmacokinetic study [56] in humans did not establish a maximum tolerated dose.

## Electronic supplementary material

ESM 1 (DOCX 60 kb)

## Abbreviations

CI: Combination index
COX: Cyclooxygenase
FLAP: 5-lipoxygenase activating protein
HETE: Hydroxyeicosatetraenoic acid
IHC: Immunohistochemistry
LOX: Lipoxygenase
LT: Leukotriene
MPM: Malignant pleural mesothelioma
NSCLC: Non small cell lung cancer
PGE_2_: Prostaglandin E_2_

## References

[1] Lanphear BP, Buncher CR (1992). Latent period for malignant mesothelioma of occupational origin. J Occup Med.

[2] Carbone M, Kratzke RA, Testa JR (2002). The pathogenesis of mesothelioma. Semin Oncol.

[3] Ceresoli GL, Locati LD, Ferreri AJ (2001). Therapeutic outcome according to histologic subtype in 121 patients with malignant pleural mesothelioma. Lung Cancer.

[4] O’Kane SL, Cawkwell L, Campbell A, Lind MJ (2005). Cyclooxygenase-2 expression predicts survival in malignant pleural mesothelioma. Eur J Cancer.

[5] Vogelzang NJ, Rusthoven JJ, Symanowski J, Denham C, Kaukel E, Ruffie P, Gatzemeier U, Boyer M, Emri S, Manegold C, Niyikiza C, Paoletti P (2003). Phase III study of pemetrexed in combination with cisplatin versus cisplatin alone in patients with malignant pleural mesothelioma. J Clin Oncol.

[6] Tsao AS, Wistuba I, Roth JA, Kindler HL (2009). Malignant pleural mesothelioma. J Clin Oncol.

[7] Ceresoli GL, Zucali PA, Gianoncelli L, Lorenzi E, Santoro A (2010). Second-line treatment for malignant pleural mesothelioma. Cancer Treat Rev.

[8] Grosso F, Scagliotti GV (2012). Systemic treatment of malignant pleural mesothelioma. Future Oncol.

[9] Clària J, Romano M (2005). Pharmacological intervention of cyclooxygenase-2 and 5-lipoxygenase pathways. Impact on inflammation and cancer. Curr Pharm Des.

[10] Greenhough A, Smartt HJM, Moore AE, Roberts HR, Williams AC, Paraskeva C, Kaidi A (2009). The COX-2/PGE2 pathway: key roles in the hallmarks of cancer and adaptation to the tumour microenvironment. Carcinogenesis.

[11] Salvado MD, Alfranca A, Haeggström JZ, Redondo JM (2012). Prostanoids in tumor angiogenesis: therapeutic intervention beyond COX-2. Trends Mol Med.

[12] Goossens L, Pommery N, Hénichart JP (2007). COX-2/5-LOX dual acting anti-inflammatory drugs in cancer chemotherapy. Curr Top Med Chem.

[13] Schneider C, Pozzi A (2011). Cyclooxygenases and lipoxygenases in cancer. Cancer Metastasis Rev.

[14] Fu S, Wu Y, Zhang Y, Qiao MM, Chen Y (2004). Anti-cancer effects of COX-2 inhibitors and their correlation with angiogenesis and invasion in gastric cancer. World J Gastroenterol.

[15] Cerella C, Sobolewski C, Chateauvieux S, Henry E, Schnekenburger M, Ghelfi J, Dicato M, Diederich M (2011). COX-2 inhibitors block chemotherapeutic agent-induced apoptosis prior to commitment in hematopoietic cancer cells. Biochem Pharmacol.

[16] Chan AT, Giovannucci EL, Meyerhardt JA, Schernhammer ES, Curhan GC, Fuchs CS (2005). Long-term use of aspirin and nonsteroidal anti-inflammatory drugs and risk of colorectal cancer. JAMA.

[17] O’Kane SL, Eagle GL, Greenman J, Lind MJ, Cawkwell L (2010). COX-2 specific inhibitors enhance the cytotoxic effects of pemetrexed in mesothelioma cell lines. Lung Cancer.

[18] Orlando UD, Garona J, Ripoll GV, Maloberti PM, Solano ÁR, Avagnina A, Gomez DE, Alonso DF, Podestá EJ (2012). The functional interaction between acyl-coa synthetase 4, 5-lipooxygenase and cyclooxygenase-2 controls tumor growth: a novel therapeutic target. PLoS One.

[19] Moore Gillian, Pidgeon Graham (2017). Cross-Talk between Cancer Cells and the Tumour Microenvironment: The Role of the 5-Lipoxygenase Pathway. International Journal of Molecular Sciences.

[20] Wang D, DuBois R (2010). Eicosanoids and cancer. Nat Rev Cancer.

[21] Tong W-G, Ding X-Z, Talamonti MS, Bell RH, Adrian TE (2005). LTB4 stimulates growth of human pancreatic cancer cells via MAPK and PI-3 kinase pathways. Biochem Biophys Res Commun.

[22] Ihara A, Wada K, Yoneda M, Fujisawa N, Takahashi H, Nakajima A (2007). Blockade of leukotriene B4 signaling pathway induces apoptosis and suppresses cell proliferation in colon cancer. J Pharmacol Sci.

[23] Kandouz M, Nie D, Pidgeon GP, Krishnamoorthy S, Maddipati KR, Honn KV (2003). Platelet-type 12-lipoxygenase activates NF-kappaB in prostate cancer cells. Prostaglandins Other Lipid Mediat.

[24] Honn KV, Timár J, Rozhin J, Bazaz R, Sameni M, Ziegler G, Sloane BF (1994). A lipoxygenase metabolite, 12-(S)-HETE, stimulates protein kinase C-mediated release of cathepsin B from malignant cells. Exp Cell Res.

[25] Hong SH, Avis I, Vos MD, Martínez A, Treston AM, Mulshine JL (1999). Relationship of arachidonic acid metabolizing enzyme expression in epithelial cancer cell lines to the growth effect of selective biochemical inhibitors. Cancer Res.

[26] Hennig R, Ding X-Z, Tong W-G, Schneider MB, Standop J, Friess H, Büchler MW, Pour PM, Adrian TE (2002). 5-lipoxygenase and leukotriene B(4) receptor are expressed in human pancreatic cancers but not in pancreatic ducts in normal tissue. Am J Pathol.

[27] Gupta S, Srivastava M, Ahmad N, Sakamoto K, Bostwick DG, Mukhtar H (2001). Lipoxygenase-5 is overexpressed in prostate adenocarcinoma. Cancer.

[28] Yoshimura R, Matsuyama M, Tsuchida K (2003). Expression of lipoxygenase in human bladder carcinoma and growth inhibition by its inhibitors. J Urol.

[29] Jiang WG, Douglas-Jones A, Mansel RE (2003). Levels of expression of lipoxygenases and cyclooxygenase-2 in human breast cancer. Prostaglandins Leukot Essent Fat Acids.

[30] Öhd JF, Nielsen CK, Campbell J, Landberg G, Löfberg H, Sjölander A (2003). Expression of the leukotriene D4 receptor CysLT1, COX-2, and other cell survival factors in colorectal adenocarcinomas. Gastroenterology.

[31] Winer I, Normolle DP, Shureiqi I, Sondak VK, Johnson T, Su L, Brenner DE (2002). Expression of 12-lipoxygenase as a biomarker for melanoma carcinogenesis. Melanoma Res.

[32] Chen X, Wang S, Wu N, Sood S, Wang P, Jin Z, Beer DG, Giordano TJ, Lin Y, Shih WC, Lubet RA, Yang CS (2004). Overexpression of 5-lipoxygenase in rat and human esophageal adenocarcinoma and inhibitory effects of Zileuton and celecoxib on carcinogenesis. Clin Cancer Res.

[33] Hoque A, Lippman SM, Wu T-TT (2005). Increased 5-lipoxygenase expression and induction of apoptosis by its inhibitors in esophageal cancer: a potential target for prevention. Carcinogenesis.

[34] Li N, Sood S, Wang S, Fang M, Wang P, Sun Z, Yang CS, Chen X (2005). Overexpression of 5-lipoxygenase and cyclooxygenase 2 in hamster and human oral cancer and chemopreventive effects of zileuton and celecoxib. Clin Cancer Res.

[35] Romano M, Catalano A, Nutini M (2001). 5-lipoxygenase regulates malignant mesothelial cell survival: involvement of vascular endothelial growth factor. FASEB J.

[36] Cawkwell L, Gray S, Murgatroyd H, Sutherland F, Haine L, Longfellow M, O'Loughlin S, Cross D, Kronborg O, Fenger C, Mapstone N, Dixon M, Quirke P (1999). Choice of management strategy for colorectal cancer based on a diagnostic immunohistochemical test for defective mismatch repair. Gut.

[37] Steinhilber D, Hofmann B (2014). Recent advances in the search for novel 5-lipoxygenase inhibitors. Basic Clin Pharmacol Toxicol.

[38] Evans JF, Ferguson AD, Mosley RT, Hutchinson JH (2008). What’s all the FLAP about?: 5-lipoxygenase-activating protein inhibitors for inflammatory diseases. Trends Pharmacol Sci.

[39] Deschamps JD, Kenyon VA, Holman TR (2006). Baicalein is a potent in vitro inhibitor against both reticulocyte 15-human and platelet 12-human lipoxygenases. Bioorg Med Chem.

[40] DeWitt DL (1999). Cox-2-selective inhibitors: the new super aspirins. Mol Pharmacol.

[41] Albrecht W, Unger A, Nussler AK, Laufer S (2008). In vitro metabolism of 2-[6-(4-chlorophenyl)-2,2-dimethyl-7-phenyl-2,3-dihydro-1H-pyrrolizin-5-yl] acetic acid (licofelone, ML3000), an inhibitor of cyclooxygenase-1 and -2 and 5-lipoxygenase. Drug Metab Dispos.

[42] Chou T-C, Talalay P (1984). Quantitative analysis of dose-effect relationships: the combined effects of multiple drugs or enzyme inhibitors. Adv Enzym Regul.

[43] Edwards JG, Faux SP, Plummer SM (2002). Cyclooxygenase-2 expression is a novel prognostic factor in malignant mesothelioma n. Clin Cancer Res.

[44] Baldi A (2004). Prognostic significance of cyclooxygenase-2 (COX-2) and expression of cell cycle inhibitors p21 and p27 in human pleural malignant mesothelioma. Thorax.

[45] Mineo TC, Ambrogi V, Cufari ME, Pompeo E (2010). May cyclooxygenase-2 (COX-2), p21 and p27 expression affect prognosis and therapeutic strategy of patients with malignant pleural mesothelioma?. Eur J Cardiothorac Surg.

[46] Nuvoli B, Galati R (2013). Cyclooxygenase-2, epidermal growth factor receptor, and aromatase signaling in inflammation and mesothelioma. Mol Cancer Ther.

[47] Lee HZ, Leung HWC, Lai MY, Wu CH (2005). Baicalein induced cell cycle arrest and apoptosis in human lung squamous carcinoma CH27 cells. Anticancer Res.

[48] Chao J-I, Su W-C, Liu H-F (2007). Baicalein induces cancer cell death and proliferation retardation by the inhibition of CDC2 kinase and survivin associated with opposite role of p38 mitogen-activated protein kinase and AKT. Mol Cancer Ther.

[49] Takahashi H, Chen MC, Pham H, Angst E, King JC, Park J, Brovman EY, Ishiguro H, Harris DM, Reber HA, Hines OJ, Gukovskaya AS, Go VLW, Eibl G (2011). Baicalein, a component of Scutellaria baicalensis, induces apoptosis by Mcl-1 down-regulation in human pancreatic cancer cells. Biochim Biophys Acta.

[50] Chen K, Zhang S, Ji Y, Li J, An P, Ren H, Liang R, Yang J, Li Z (2013). Baicalein inhibits the invasion and metastatic capabilities of hepatocellular carcinoma cells via down-regulation of the ERK pathway. PLoS One.

[51] Tavolari S, Bonafè M, Marini M, Ferreri C, Bartolini G, Brighenti E, Manara S, Tomasi V, Laufer S, Guarnieri T (2008). Licofelone, a dual COX/5-LOX inhibitor, induces apoptosis in HCA-7 colon cancer cells through the mitochondrial pathway independently from its ability to affect the arachidonic acid cascade. Carcinogenesis.

[52] Fischer AS, Metzner J, Steinbrink SD, Ulrich S, Angioni C, Geisslinger G, Steinhilber D, Maier TJ (2010). 5-lipoxygenase inhibitors induce potent anti-proliferative and cytotoxic effects in human tumour cells independently of suppression of 5-lipoxygenase activity. Br J Pharmacol.

[53] Aryal P, Kim K, Park P-H, Ham S, Cho J, Song K (2014). Baicalein induces autophagic cell death through AMPK/ULK1 activation and downregulation of mTORC1 complex components in human cancer cells. FEBS J.

[54] Melstrom LG, Bentrem DJ, Salabat MR, Kennedy TJ, Ding XZ, Strouch M, Rao SM, Witt RC, Ternent CA, Talamonti MS, Bell RH, Adrian TA (2008). Overexpression of 5-lipoxygenase in colon polyps and cancer and the effect of 5-LOX inhibitors in vitro and in a murine model. Clin Cancer Res.

[55] Timár J, Rásó E, Döme B, Li L, Grignon D, Nie D, Honn KV, Hagmann W (2000). Expression, subcellular localization and putative function of platelet-type 12-lipoxygenase in human prostate cancer cell lines of different metastatic potential. Int J Cancer.

[56] Li M, Shi A, Pang H, Xue W, Li Y, Cao G, Yan B, Dong F, Li K, Xiao W, He G, Du G, Hu X (2014). Safety, tolerability, and pharmacokinetics of a single ascending dose of baicalein chewable tablets in healthy subjects. J Ethnopharmacol.

