# Supplementary material for: The Investigation of Lipoxygenases as Therapeutic Targets in Malignant Pleural Mesothelioma
Source: Pathol Oncol Res. 2019 Apr 2;26(2):985–95. doi: 10.1007/s12253-019-00652-x (PMC7242492; doi:10.1007/s12253-019-00652-x)
Supplement: Supplementary file 1 — (DOCX 60 kb) [file 12253_2019_652_MOESM1_ESM.docx]

**The investigation of lipoxygenases as therapeutic targets in malignant pleural mesothelioma**

Lily OGUH-OLAYINKA^1*^, Vijay AGARWAL^1, 2^, Dulani RANATUNGE^1^, Anne CAMPBELL^3^, Stefan LAUFER^4^, Lynn CAWKWELL^1,5^, Michael J. LIND^1,2^.

^1^Hull York Medical School, Hull, UK.

^2^Queens Centre for Oncology and Haematology, Hull and East Yorkshire NHS Trust, Hull, UK.

^3^Histopathology Department, Hull and East Yorkshire NHS Trust, Hull, UK.

^4^Department of Pharmaceutical Chemistry, Eberhard Karls University, Tübingen, Germany.

^5^Department of Biomedical Science, University of Hull, Hull, UK.

*Corresponding author:

Dr Lily Oguh-Olayinka PhD, Research Laboratories, Daisy Building, Castle Hill Hospital, Hull, HU16 5JQ, UK.

Tel: +44 (0)1482 461850; Fax +44 (0)1482 461874

E-mail: [L.Cawkwell@hull.ac.uk](mailto:L.Cawkwell@hull.ac.uk)

| **A** | 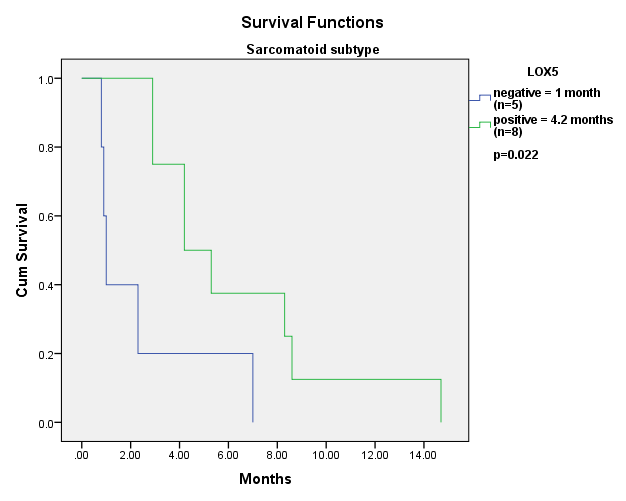 | **B** | 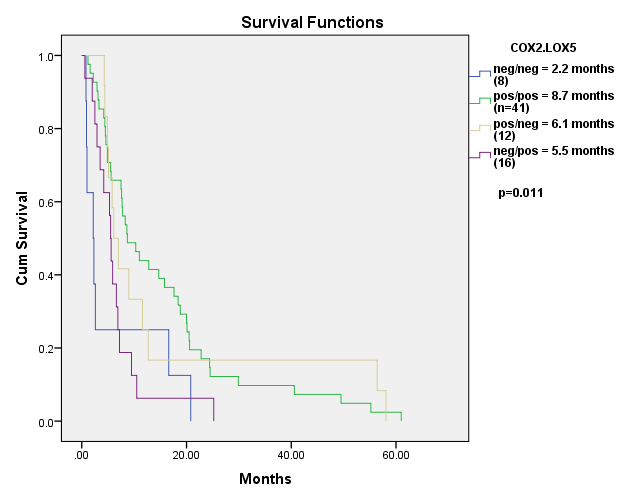 |
| --- | --- | --- | --- |
| **C** | 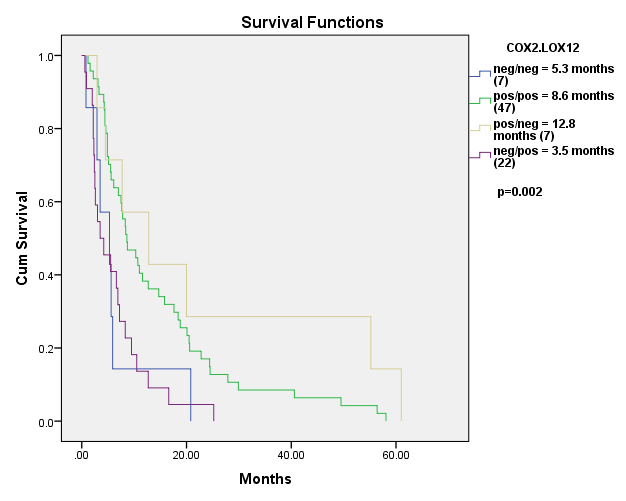 |  |  |

**Online Resource 1: Kaplan Meier survival analyses. A: Kaplan Meier survival analysis for 5-LOX protein expression in the sarcomatoid subtype (n = 13).** Median survival was 4.2 months in 5-LOX positive cases (green line) *versus* 1 month in 5-LOX negative cases (blue line) for the sarcomatoid subtype (p = 0.022, log rank). **B: Kaplan Meier** **survival analysis for the co-expression of COX-2 and 5-LOX proteins.** Median survival was 8.7 months in cases demonstrating co-expression of COX-2/5-LOX (green line) *versus* 2.2 months in cases demonstrating negative expression of both proteins (blue line) (p = 0.011, log rank). **C: Kaplan Meier** **survival analysis for the co-expression of COX-2 and 12-LOX proteins.** Median survival was 12.8 months in cases demonstrating the COX-2 positive /12-LOX negative status (yellow line) *versus* 3.5 months in cases demonstrating the COX-2 negative /12-LOX positive status (purple line) (p = 0.002, log rank). Median survival was 8.6 months in cases demonstrating co-expression of COX-2/12-LOX (green line).
